# Flavone‐associated resistance of two *Lemna* species to duckweed weevil attack

**DOI:** 10.1002/ece3.9459

**Published:** 2022-11-18

**Authors:** Gisuk Lee, Hanyoung Choi, Youngsung Joo, Sang‐Gyu Kim

**Affiliations:** ^1^ Department of Biological Sciences Korea Advanced Institute for Science and Technology Daejeon Korea; ^2^ Department of Biological Sciences and Biotechnology Chungbuk National University Cheongju Korea

**Keywords:** aquatic plant, duckweed, duckweed weevil, plant defense

## Abstract

*Lemna perpusilla* and *Lemna minor* are free‐floating plants that often live in the same habitat. However, little is known about how they differ in response to herbivore attacks. In this study, we examined the species‐specific resistance of two *Lemna* species to the duckweed weevil, *Tanysphyrus lemnae*. The female adults of *T. lemnae* preferred to lay eggs on *L. perpusilla* over *L. minor*. In addition, the larvae of *T. lemnae* performed better when fed on *L. perpusilla* than on *L. minor*. To understand the physiological basis of species‐specific resistance in the two *Lemna* species, we measured the amounts of jasmonic acid (JA), phytosterols, and flavonoids. Attacks by duckweed weevils increased the levels of JA in the two *Lemna* species, but these levels did not differ significantly between the two species. Interestingly, the levels of flavones (isoorientin, vitexin, and isovitexin) in *L. minor* species were higher than those in *L. perpusilla*. The in vitro bioassay showed that three flavones significantly decreased the survival rate of duckweed weevil larvae. Although *L. perpusilla* was less resistant to duckweed weevil attack compared to *L. minor*, *L. perpusilla* grew faster than *L. minor* regardless of the duckweed weevil attack. These results suggest that these two *Lemna* species have different defense strategies against the duckweed weevil.

## Graphical abstract

*Lemna perpusilla* and *Lemna minor* are free‐floating plants that often live in the same habitat. Two aquatic *Lemna* species make different growth‐defense tradeoffs against herbivore attack. *L. minor* accumulates more toxic compounds that function by decreasing the larval survival rates.

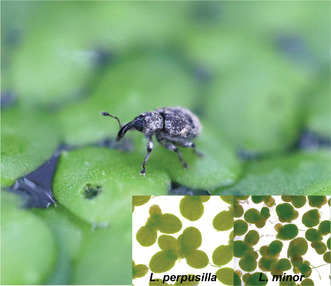

## 1 INTRODUCTION

Plants interact with herbivorous insects in various ecosystems. Most studies have involved terrestrial plants and herbivores because interactions in terrestrial ecosystems are regarded as more diverse and abundant than those in aquatic ecosystems (Forister et al., 2015; Hay & Fenical, 1988; Vermeij, 2016). Although the interaction between aquatic plants and herbivorous insects is poorly understood (Vermeij, 2016), herbivorous insects are known to negatively affect plant abundance in aquatic habitats (Bakker et al., 2016; Wood et al., 2017). For instance, herbivorous insects in aquatic environments reduce plant growth and viability (Doyle et al., 2007; Owens et al., 2006). In addition, aquatic herbivores affect aquatic restoration, plant reproduction, and plant competition in aquatic environments (Harms et al., 2011; Van et al., 1998). Therefore, it is necessary to understand the interaction between aquatic plants and herbivorous insects at the community level.

Aquatic herbivorous insects integrate many factors when selecting a host plant for oviposition and offspring feeding (Cherian et al., 2001; Scheirs & Bruyn, 2000; Thompson, 1988); the levels of primary as well as secondary metabolites are key factors for host selection (Anderson et al., 2011; Awmack & Leather, 2002). For instance, the phenolic secondary metabolites of aquatic plants were negatively correlated with herbivore feeding preferences (Lodge, 1991; Vergeer & Van Der Velde, 1997). Flavonoids play various roles in plants. Flavonoids regulate plant growth and photosynthesis, and they protect cells from UV‐damage, drought, and pathogen and herbivore attacks (Pagliuso et al., 2020; Pichersky & Gang, 2000; Yonekura‐Sakakibara et al., 2019). The defense‐related flavonoids can be divided into two categories: the inducible flavonoids, which are induced by abiotic and biotic stresses, and the constitutive flavonoids, which are synthesized during normal development (Treutter, 2005). The constitutive metabolites are stored in strategically important tissues, such as flowers and fruits (Brunetti et al., 2013; Taylor & Grotewold, 2005). Some flavonoid metabolites of the aquatic fern *Azolla pinnata* act as chemical deterrents to aquatic herbivores (Cohen et al., 2002). In addition, submerged *Elodea* plants contain flavone glucosides, which reduce the feeding preference and growth of aquatic herbivorous Lepidoptera (Erhard et al., 2007). In the case of phytohormones, several studies mentioned the possible role of phytohormones in Lemnaceae family. For instance, exogenously applied JA (jasmonic acid) and ABA (abscisic acid) at low concentrations were found to induce flowering in *Spirodela polyrrhiza* and *Lemna minor* (Krajncic et al., 2006; Piotrowska et al., 2010). Another study has tested the effect of exogenous phytohormones (SA; salicylic acid, ABA and JA so on) in the growth regulation of *L. minor* (Utami et al., 2018). Most studies focused on the effect of the exogenous application of phytohormones but not the endogenous function of phytohormone in *Lemna* species.

In nature, plants have to cope with various biotic stresses and constantly face attacks by pathogens and herbivorous insects (Baldwin, 2001; Walling, 2009). In addition, plants need to balance growth and defense to optimize their fitness (Huot et al., 2014). Growth‐defense tradeoffs drive differential intra‐ or inter‐species competition and affect the susceptibility of host plants to herbivore attack (de Vries et al., 2017). Because many aquatic plants grow fast, aquatic plants can be a useful model system for studying growth‐biased strategies in the growth‐defense trade‐off phenomenon in plants (Acosta et al., 2021).

Lemnaceae are the smallest and fastest‐growing flowering plants (Kurepa et al., 2018; Laird & Barks, 2018). Duckweeds have a leaf‐like structure called a frond (or thallus), no stem, and one or more roots. Although duckweeds can produce flowers, they normally propagate vegetatively (Hillman, 1961). Duckweeds comprise five genera (*Spirodela*, *Landoltia*, *Lemna*, *Wolffiella*, and *Wolffia*) and 36 species in the world (Acosta et al., 2021). Species of the common duckweed, *Lemna*, are distributed globally (Silva et al., 2018). Among the genus *Lemna*, *L. minor* has been extensively studied, because *L. minor* plants are also used for phytoremediation and dietary supplements (Wang et al., 2016). Moreover, small genome sizes, genetic manipulation techniques that enable functional testing of genes, and rapid vegetative propagation make it suitable for a variety of research applications including biochemistry, metabolism, and interactions with microbial communities (Acosta et al., 2021). The preferences of water‐lily aphid preferences for four duckweed species were examined recently, but it has not been tested mechanism of preference of the aphid (Subramanian & Turcotte, 2020). On the other hand, most studies on the chemical side of *Lemna* species focused on metabolic changes (e.g., starch, phenolics and flavonoids) in responses to abiotic stress (Pagliuso et al., 2020; Tao et al., 2017). Despite their central roles in aquatic ecosystems and their heavy use in biotechnology, the defense responses of *Lemna* species to herbivorous insects are still poorly understood.

The purpose of this study was to examine how two aquatic free‐floating plants, *L. perpusilla* and *L. minor*, defend against a duckweed weevil, *Tanysphyrus lemnae*, attack. We hypothesized that two *Lemna* species have different chemical defenses against herbivores. In order to test this hypothesis, we first examined the duckweed weevil's oviposition preference and larval performance in two *Lemna* species. Phytohormones and metabolites were then measured in two *Lemna* species in response to weevil attacks. Lastly, we examined the defensive role of metabolites and the growth‐defense trade‐off in both *Lemna* species.

## 2 MATERIALS AND METHODS

### 2.1 Plant and insect materials and growth conditions

Wild‐type *L. perpusilla* (minute duckweed) was collected from a natural population in Daejeon, South Korea. *L. minor* (common duckweed) was obtained from the biological resource center of the Korea Research Institute of Bioscience and Biotechnology (KRIBB, Jeonbuk, South Korea), and was originally collected from Jeju island, South Korea. We propagated *L. perpusilla* and *L. minor* plants from a single colony. Sterilized colonies were grown in fertilizer solution (Kinnersley & Lin, 2000) for experiments and insect colony maintenance.

**FIGURE 1:**
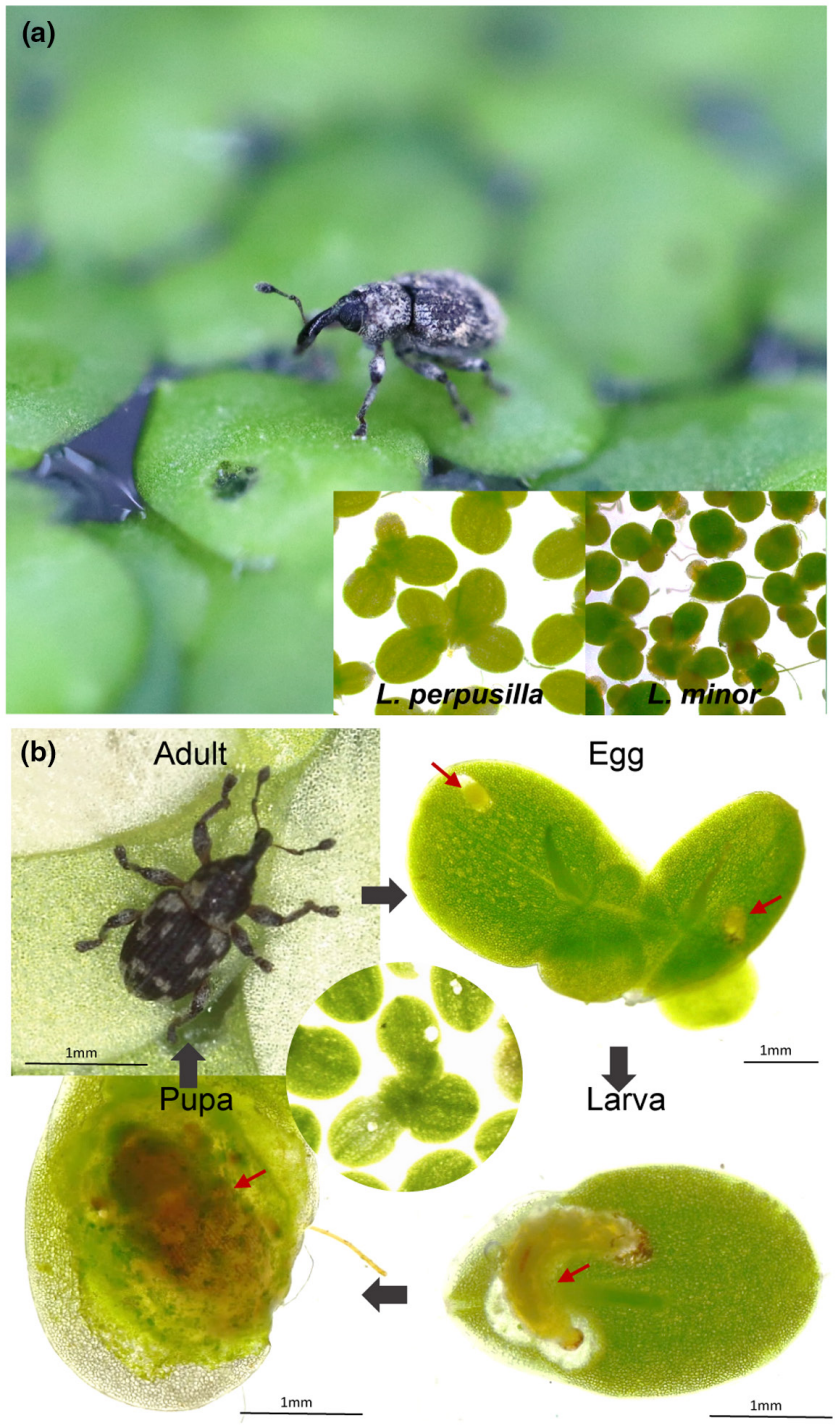
Aquatic duckweed weevil, *Tanysphyrus lemnae* feeds on two *Lemna* species in a native habitat (a) *T. lemnae* adults, *L. perpusilla*, and *L. minor* were collected from their native habitat. (b) Developmental stages of the duckweed weevil, *T. lemnae*. The female deposits one egg (red arrow) per frond. After 5–7 days, an egg hatches (red arrow) inside a frond, and a larva starts to feed, moving freely to a new frond when needed. After about 2 weeks, the larva begins to pupate (red arrow) inside of the frond. An adult emerges after 7 days.

*Lemna perpusilla* and *L. minor* are distributed in the same habitats, such as wetlands, slow‐flowing streams, upper estuaries, paddy fields, agricultural waterways, lakes, and ponds, in South Korea. During maturation, the daughter fronds of *Lemna* species are launched from the axial meristematic zone of the mother frond. The central position of the daughter–mother frond is connected by a stipule; this stipule breaks off after maturation (Cherian et al., 2001; Topp et al., 2011). To differentiate between the two sibling species, we confirmed that the frond of *L. perpusilla* has a winged root sheath at its base, an ovate shape, thalli without anthocyanin pigment, and a lighter green leaf color than the frond of *L. minor* (Hillman, 1961; Landolt, 1986) (Figure 1a). All plants were grown at 25 ± 2°C, 16 h light/ 8 h dark cycle with 60% relative humidity and 100 μmol m^−2^ s^−1^ of white light in the growth room.

*Tanysphyrus lemnae* is a weevil of aquatic herbivorous insect distributed native to Europe to North America and occurs through Asia and Japan. *Tanysphyrus lemnae* refers to the host plant, *Lemna* genera (Paykull, 1792). Adult weevils chew on the fronds using chewing mouthparts located at the end of long snouts, creating round holes. The larvae of *T. lemnae* also feed on the fronds, but the larvae tunnel through the frond in curved patterns like miners. We collected adult duckweed weevils of *T. lemnae* from the same pond in which *L. perpusilla* plants were collected (Figure 1a). We developed *T. lemane* weevil colony in laboratory conditions for further experiments. The adults were reared in a plastic cage with a ventilation hole that was covered with a nylon mesh (diameter 12 × height 8 cm, hole 4 cm, insect breeding dish, SPL, South Korea) and allowed to mating and ovipositing. *Tanysphyrus lemnae* females laid one egg on the fronds' abaxial (lower) part per one frond. The fronds with an oviposited egg were collected and placed in a new cage. One week after moving (ovipositing), the eggs hatched and the larvae began feeding on the intact frond for a week. After that the larvae became pupation stage. Newly adults hatched five to seven days later, (Figure 1b). Insects were maintained under the same conditions as duckweed.

### 2.2 Adult preference and larval performance

Adult preference was estimated by measuring the feeding area and by counting eggs in plastic cages same as the insect colony cage (diameter 12 × height 8 cm, SPL, South Korea) that contained two compartments. We placed the same area of the two *Lemna* species, *L. perpusilla* and *L. minor* to eliminate feeding and oviposition biases caused by differences in frond areas (10 *L. perpusilla* trifoliate fronds and 15 *L. minor* trifoliate fronds). One gravid female weevil was placed in the middle of each cage. For the preference test, we placed the two *Lemna* species in each custom‐made small plastic plate (diameter 40 mm x height 8 cm) with the central part removed for easier floating. Eighteen cages were used for the preference assay. The two *Lemna* species were randomly placed to avoid positional effects. We first allowed newly born females to mate for five days, and then released the mated females for three days. After that, we measured the area of fronds that had been consumed by the female feeding using ImageJ software, and we counted the number of eggs oviposited on fronds using a microscope (SMZ645, Nikon).

To examine the larval performance of *T. lemnae* species, we measured the length of larvae feeding on each species of *L. perpusilla* and *L. minor* species. Each 500 fronds of *L. perpusilla* and *L. minor* species were placed in each plastic cage (L 21 × W 21 × H 6 cm, ventilation hole diameter 10 cm with 300 μm aperture mesh, BugDorm, MeagView Science Co.) for a sufficient amount of food was supplied during the experiment period. Duckweed weevils (25 males and 25 females) were released for mating and ovipositing in the cage, then removed after 1 day. After 7 days, we collected larvae. To quantify the length of larvae, 10 larvae were collected each from *L. perpusilla* and *L. minor* and took pictures to process the images with ImageJ software (1.53 e, National Institutes of Health).

### 2.3 Phytohormone analysis

Six‐pooled frond samples were collected from each duckweed species, both intact and damaged by *T. lemnae* adults for an hour. There are six biological replicates. The method of phytohormone extraction (JA; jasmonic acid, SA; salicylic acid and ABA; abscisic acid) and quantification were previously described in Joo et al. (2021). Briefly, approximately 100 mg of each frozen frond sample was homogenized with two steel beads in a TissueLyser II (Qiagen) for 1 min at 26 Hz after adding 1 ml ethyl acetate spiked with internal standards mixture: 20 ng each of [^2^H_2_] JA, [^2^H_4_] SA and [^2^H_6_] ABA. The extracted samples were centrifuged at 16,100 *g* at 4°C for 20 min, and the supernatant was transferred into another new tube. The samples were evaporated to near dryness in a centrifugal vacuum concentrator (VC2124, Gyrogen) at 30°C. The dried samples were dissolved in 500 μl 70% (v/v) methanol: water for analysis with ultra‐high performance liquid chromatography (UHPLC) triple‐quadrupole mass spectrometry (LC–MS‐8050, Shimadzu) as described previously (Joo et al., 2021). The phytohormones were detected in negative electrospray ionization mode (ESI), and the detailed detection method followed by Schäfer et al. (2016). The amounts of phytohormones were normalized by dividing the peak area of each phytohormone by the exact fresh mass of plant materials and the internal standards of each phytohormone.

### 2.4 Phytosterol and primary metabolite analysis

We followed the extraction procedure described by Suh et al. (2013) for the phytosterols (campesterol, stigmasterol, and beta‐sitosterol) and primary metabolite analysis. There are six biological replicates. In each of the two *Lemna* samples, 10 mg was freeze‐dried and extracted with 1 ml of methanol (HPLC grade, Sigma). The samples were sonicated for 40 min. The supernatants were collected and filtered through 0.45 μm PTFE syringe filters. Each sample (one from each species) of 100 μl was transferred into amber vials and dried with nitrogen gas for 5 min. For derivatization, 30 μl of 20,000 μg/ml methoxylamine hydrochloride (Sigma) in pyridine, 50 μl of N, O‐bistrifluoroacetamide containing 1% trimethylchlorosilane (Sigma), and 10 μl of 300 μg/ml 2‐chloronaphthalene (Sigma) in pyridine as an internal standard were added to each dried sample. The samples were incubated at 65°C for 1 h and analyzed by gas chromatography–mass spectrometry (GC–MS, QP2020, Shimadzu). The compounds were putatively identified using the national institute of standards and technology (NIST) mass spectral search program, and a matching similarity greater than 80% was used as the compound identity. In control and treatment samples of two *Lemna* species, each peak area of metabolites were normalized by those of the internal standard for the relative abundance of the metabolites. Using the MetaboAnalyst 4.0 platform (www.metaboanalyst.ca), normalized peak areas were calculated from the control and damaged samples for each metabolite.

### 2.5 Flavonoid analysis

To examine flavonoid compounds in the two *Lemna* species, two *Lemna* species were released into each plastic cage that was used for the insect colony maintenance. We allowed 15 adult duckweed weevils to feed on plants for 10 days. There were five biological replicates. Approximately 100 mg of frozen materials was homogenized with a steel pestle and extracted by adding 200 μl of the extraction buffer (75% methanol/ 0.1% formic acid) as described in (Gomez et al., 2018). Supernatants were collected after ultrasound treatment for 30 min followed by centrifugation at 16,000 *g* at 4°C for 30 min. The procedures were repeated twice. The collected supernatants were lyophilized for 24 h and resuspended in distilled water. The samples were stored in the freezer at −80°C until analysis. Aliquots of 300 μl of the re‐suspended samples were transferred into amber vials with an insert were analyzed by UPLC–MS/MS (LC–MS‐8050, Shimadzu), and 1 μl of the extracts was injected by the autosampler into the LC–MS system and chromatographic separation were carried out on a C18 column (UPLC BEH, 1.7 μm particle size, 100 mm length × 2.1 mm inner diameter, Waters). The solvents used in the mobile phases were deionized water containing 0.02% acetic acid (A); solvent B consisted of 0.02% acetic acid in acetonitrile, with the following concentration gradient of B: 5%, 0 min; 60%, 11 min; 95%, 13 min; 95%, 15 min; 5%, 16 min; 5%, 17 min. The mass spectrometer was operated in positive ESI mode using the same chromatographic conditions. Using Q3 scan and MRM (multiple reaction monitoring) methods, we analyzed a total of 18 target flavonoid compounds: four flavonols, six flavones, four flavanones, two chalcones, and two isoflavones (Table S1). All standard compounds were obtained from ChemFaces (Wuhan ChemFaces Biochemical Co.). Flavonoid compounds (isoorientin, vitexin, isovitexin, hesperetin, luteolin, and apigenin) were quantified by comparing their peak areas with calibration curves at a concentration of from 0.1 to 100 μg/ml.

### 2.6 In vitro bioassay of duckweed weevil larvae

In vitro bioassays were conducted using semi‐artificial diets supplemented with three flavones (isoorientin, vitexin, and isovitexin) which were the most abundant compounds in *L. minor*. We used 100 five‐day‐old larvae of *T. lemnae* that had hatched only on *L. perpusilla* plants. Each treatment consisted of 25 larvae fed an artificial diet. With individual flavones added to agar (3%) at a concentration of 100 μg/g each. The concentrations of flavones and *L. perpusilla* powder are referred to on a freeze‐dried weight basis. The concentration of flavones was similar to the maximum level of flavone compounds in *L. minor* species. In vitro assays were also performed under the same growth conditions as plant growth and insect colony maintenance. The larval survival was counted at two‐day intervals over 5 days and estimated by the Kaplan–Meier method (Kishore et al., 2010).

### 2.7 Growth performance of *Lemna* species

To examine the growth difference between two *Lemna* species, we released 30 trifoliate fronds of *L. perpusilla* and 48 trifoliate fronds of *L. minor* in each plastic cage (diameter 12 cm, height 8 cm, ventilation hole 4 cm, SPL). The sum of the frond size of 30 *L. persusilla* was similar to the sum of the frond size of 48 *L. minor*. Ten *T. lemane* weevil adults were released per cage and three cages were used for each treatment. After releasing the weevils, we took images of the damaged plants at 0, 2, 4 and 6 days. To quantify leaf areas of plants, the images were processed by ImageJ software (1.53 e, National Institutes of Health), and the damaged area by the *T. lemnae* weevil was excluded from the quantification.

### 2.8 Statistical analysis

The consumed frond area and the number of eggs deposited on each pair of the two *Lemna* species for use as choice assay and the quantified value of larvae size were analyzed by a student's *t*‐test. Metabolite contents were analyzed by two‐way ANOVA followed by Tukey's honestly significant difference (HSD) as post hoc test, and larval survival curves of treatment and control samples were compared by the log‐rank test. The total frond area to see growth differences between the two *Lemna* species was analyzed by repeated measures *t*‐test. The frequency distribution of larval preference were compared by *G*‐test. The primary metabolites profiling of two *Lemna* species was analyzed by heatmap clustering which used to calculate the Euclidean distance. Principal component analysis (PCA) was conducted using MetaboAnalyst 4.0 platform (Chong et al., 2019). Data analysis was conducted with OriginPro 2019 (OriginLab, Northampton) or the publically available R package such as psych (version 4.1.2, R Core Team, 2020).

## 3 RESULTS

### 3.1 Preference and performance of duckweed weevil, *Tanysphyrus lemnae*, that fed on two sibling *Lemna* species

**FIGURE 2:**
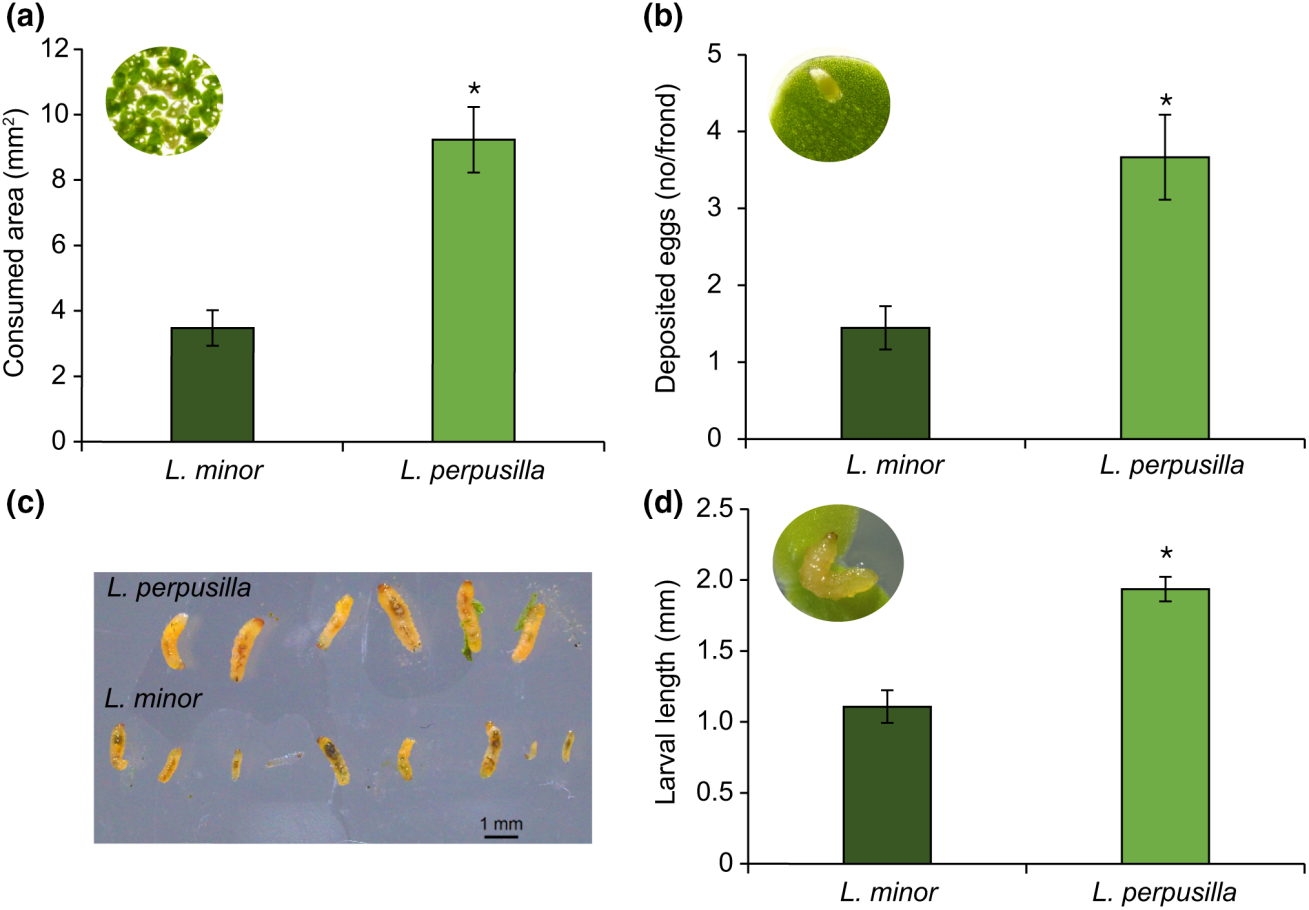
Preference and larval performance of *T. lemnae* fed on *L. perpusilla* and *L. minor* plants. (a) Frond area consumed (±SE) by *T. lemnae* adult females. (b) Number of eggs oviposited by *T. lemnae* females (±SE) (*t*‐test, *p* < .05, *n* = 18). (c) Representative larvae fed on each *L. perpusilla* (Lp) and *L. minor* (Lm) fronds for 5 days. (d) Length of *T. lemnae* larvae (±SE) fed on *L. perpusilla* and *L. minor* plants. An asterisk indicates significant differences (*t‐*test, *p* < .05, *n* = 10).

To examine the preference of duckweed weevil for two sibling *Lemna* species, we conducted dual‐choice assays that measured the insects' feeding and oviposition preferences. *Tanysphyrus lemnae* females consumed 2.65 times more fronds area of *L. perpusilla* than of *L. minor* (*p* < .05, Figure 2a). *Tanysphyrus lemnae* females also oviposited 1.74 times more eggs on fronds of *L. perpusilla* than of *L. minor* (*p* < .05, Figure 2b). To quantify larval performance, we measured the length of larvae fed on *L. perpusilla* and *L. minor* 7 days after oviposition (Figure 2c). The larvae fed on *L. perpusilla* were significantly bigger than those fed on *L. minor*. The size difference was 1.74 times more on the larval fed on *L. perpusilla* than *L. minor* (*p* < .05, Figure 2d). We further investigated larval preference and found more larvae on fronds of *L. perpusilla* than on fronds of *L. minor* (Figure S1).

### 3.2 Species‐specific defense responses of two sibling *Lemna* species to attack by duckweed weevil

**FIGURE 3:**
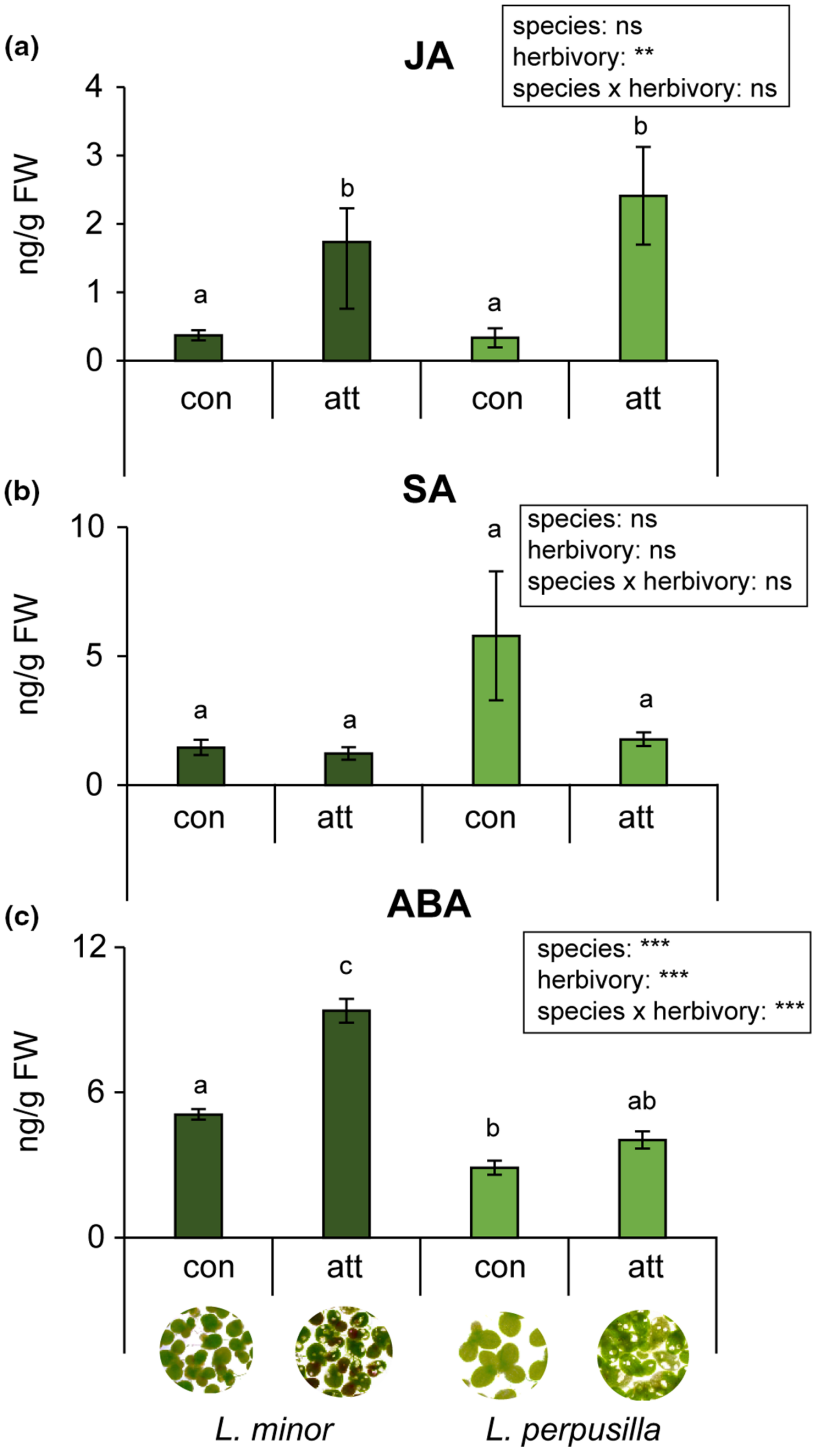
Mean (±SE) levels of jasmonic acid (JA), salicylic acid (SA), and abscisic acid (ABA) in *Lemna* species when damaged by *T. lemnae* adults. Asterisks indicate significant differences between species and treatments (**p < .01; ***p < .001, ns, no significant, *n* = 6) as determined by two‐way ANOVA analysis. Different letters indicate significant differences between two *Lemna* fronds determined by ANOVAs with post hoc tests with Tu*key* correction (con; control, damaged; damaged by the duckweed weevil, *T. lemnae*).

To understand the physiological basis of herbivore choices and performance, we measured the levels of phytohormones and plant secondary metabolites (phytosterols and flavonoids). Attack by the duckweed weevil elicited significant amounts of jasmonic acid (JA) in both *Lemna* species (*p* < .01, Figure 3a), although levels of SA (salicylic acid) stayed the same (Figure 3b). In response to adult *T. lemnae* attack, levels of ABA (abscisic acid) increased in fronds of *L. minor* but not of *L. perpusilla* and even without damaged (*p* < .001, Figure 3c).

**FIGURE 4:**
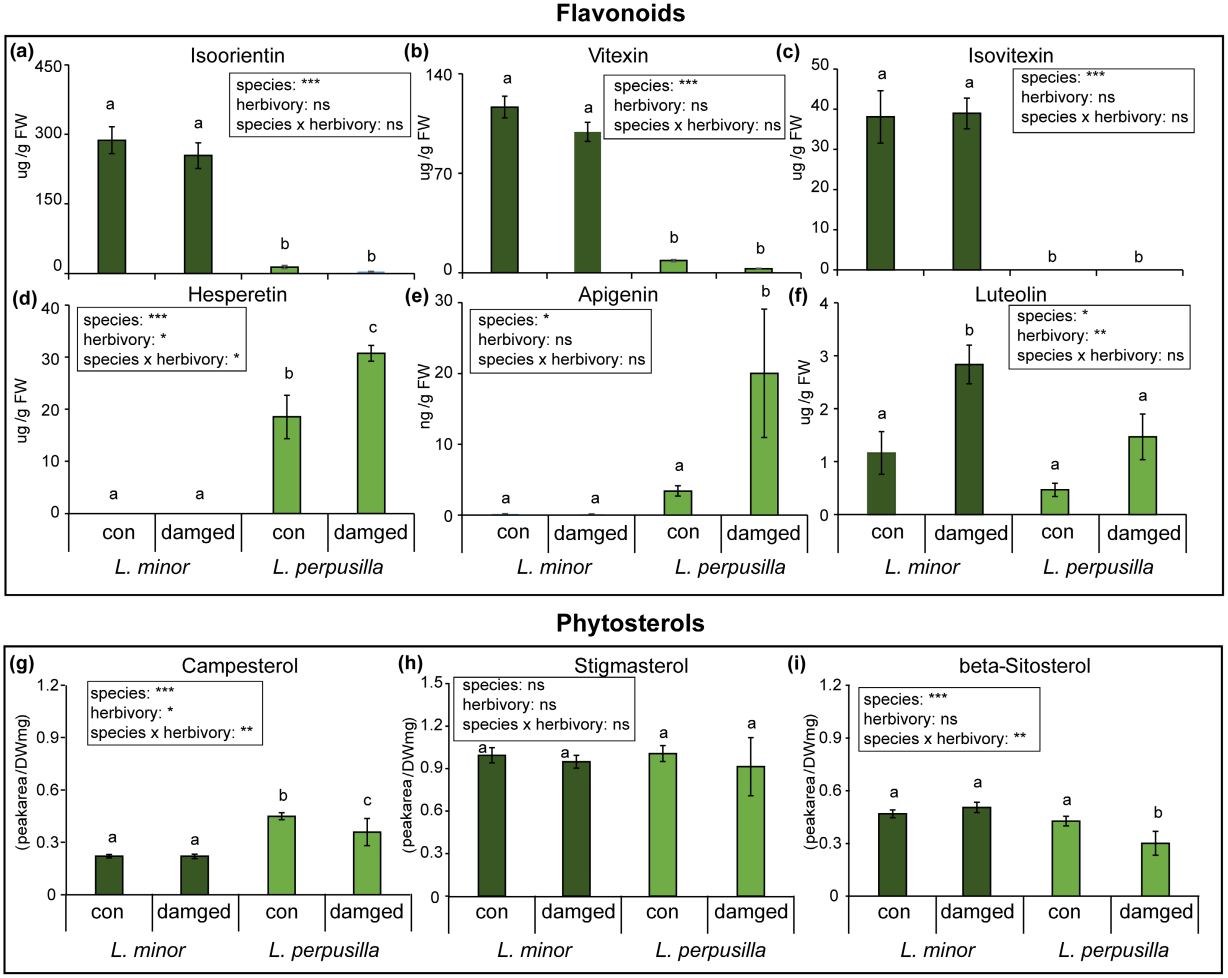
Mean (±SE) levels of flavonoids and phytosterols in *Lemna* species that had been damaged by *T. lemnae* adults. Isoorientin (a), vitexin (b), isovitexin (c), hesperetin (d), apigenin (e), and luteolin (f) were measured in *L. minor* and *L. perpusilla* plants (*n* = 5–6). The phytosterols – campestrol (g), stigmastrol (h), and beta‐sitosterol (i) – were measured in *L. minor* and *L. perpusilla* plants. Asterisks indicate significant differences between species and treatments (two‐way ANOVA, *p < .05; **p < .01; ***p < .001, ns, no significant). Different letters indicate significant differences between the two *Lemna* fronds determined by ANOVAs with post hoc tests with Tukey correction (con; control, damaged; damaged by duckweed weevil, *T. lemnae*).

Flavonoids are known to protect plants from herbivores and microbe attacks (Panche et al., 2016), so we measured their levels in two sibling *Lemna* species in response to *T. lemnae* attack. We were able to detect six flavonoid compounds in *L. perpusilla* and *L. minor* among the target list of flavonoids (Table S1). The abundance of four flavonoids (isoorientin, vitexin, isovitexin, and hesperetin) strongly differed between *L. perpusilla* and *L. minor* (Figure S2). High levels of isoorientin, vitexin, and isovitexin metabolites accumulated in both control and weevil‐attacked *L. minor* plants but not in *L. perpusilla* plants (all *p* < .001, Figure 4a–c). Meanwhile high levels of hesperetin compounds were detected in *L. perpusilla* control and damaged fronds, but not in the fronds of *L. minor* (*p* < .001, Figure 4d). Small variations were found between species or between treatments in the levels of minor flavonoid compounds, apigenin, and luteolin (all *p* < .05, Figure 4e,f). Apigenin and luteolin were elicited by herbivory in *L. perpusilla* and *L. minor*, respectively. However, flavonoid levels in duckweeds remained abundant in response to the attack by duckweed weevils (Figure 4).

In addition, we analyzed phytosterols – campesterol, stigmasterol, and *β*‐sitosterol –in two *Lemna* species. Although none of the three phytosterols were induced by herbivore attack, campesterol and *β*‐sitosterol accumulated in a species‐specific manner (Figure 4g–i, *p* < .05). We also conducted clustering analyses of primary metabolites in the *Lemna* species damaged by duckweed weevil on both sibling *Lemna* species. The heatmap analysis indicated that some primary metabolites were highly induced, especially in the attacked *L. minor* (Figure S3a). Principal component analysis (PCA) showed a differential grouping for the two *Lemna* species (Figure S3b).

### 3.3 *Lemna minor*‐specific flavones decrease larval survival of duckweed weevil

**FIGURE 5:**
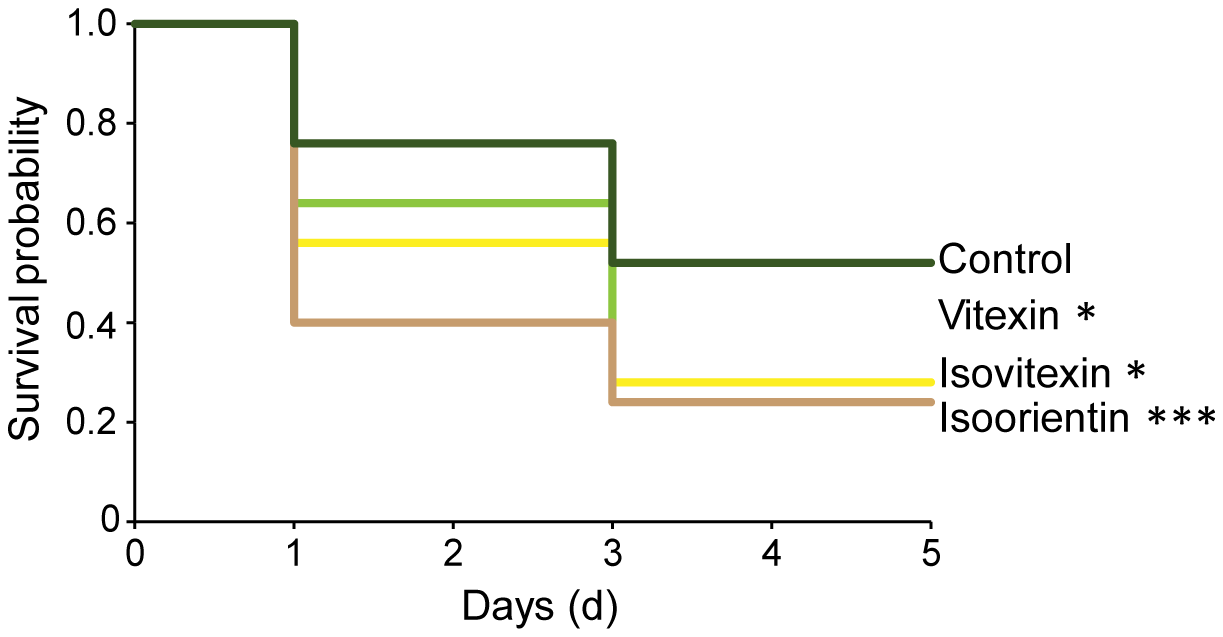
Survival curves of *T. lemnae* larvae fed on artificial diets spiked with different flavonoid compounds. Survival probability was estimated using the *Kaplan–Meier* method (*rog‐rank test*, *p < .05; ***p < .001, *n* = 25).

Because *L. minor* resistant than *L. perpusilla* to the duckweed weevil, we hypothesized that specific flavones in *L. minor* increased its resistance to *T. lemnae*. To evaluate the defensive roles of isoorientin, vitexin, and isovitexin, which accumulated mainly in *L. minor* (Figure 4), we fed the early stage of *T. lemnae* larvae on semi‐artificial diets spiked with each of these compounds. Because *L. perpusilla* barely produced any amount of these, we mixed freeze‐dried *L. perpusilla* powder with agar to make the diet. Analysis of larval survival using the Kaplan–Meier method indicated that the level of all three *L. minor*‐specific flavones significantly decreased the survival rate of duckweed larvae (log‐rank test, Figure 5).

### 3.4 The growth of the sibling *Lemna* species differed in response to herbivore attack

**FIGURE 6:**
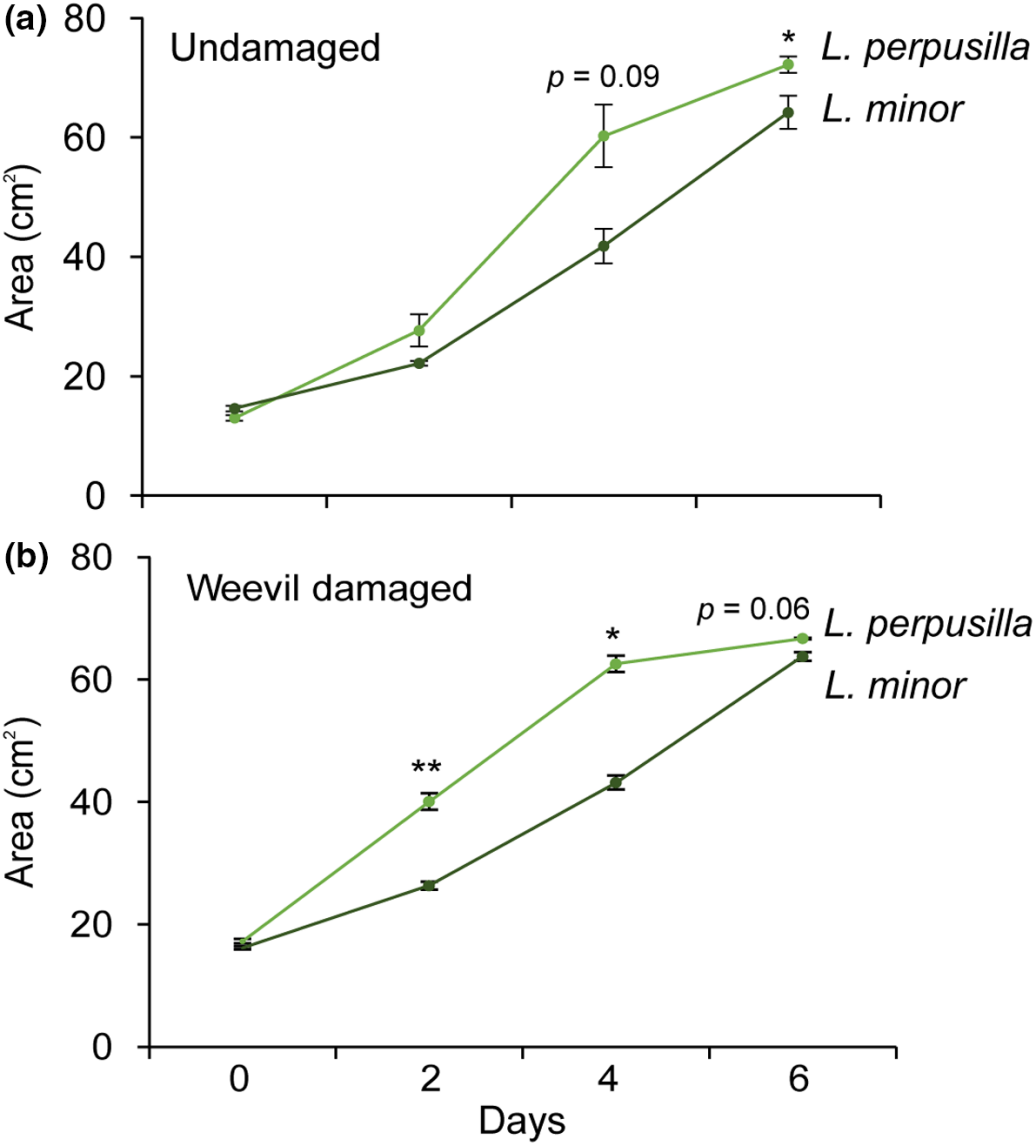
Growth difference of two sibling *Lemna* species. Mean frond area (±SE) of control and damaged *Lemna* species. An asterisk indicates significant differences (repeated *t*‐test, *p < .05; **p < .01, *n* = 3).

Next, we compared the frond area of two *Lemna* species with or without *T. lemnae* treatment. We placed 30 trifoliate fronds of *L. perpusilla* and 48 trifoliate fronds of *L. minor* in each plastic cage (Day 0) and measured the total area of fronds three times at two‐day intervals for 6 days. Significant difference was found in the total area of fronds between *L. perpusiila* and *L. minor* without herbivore attacks 6 days after release; *L. perpusilla* fronds grew faster than *L. minor* fronds (*p* < .05, Figure 6a). Fronds of *L. perpusilla* grew faster than those of *L. minor* between 2 and 4 days against duckweed weevil attacks (*p* < .01 and *p* < .05, Figure 6b). The value of frond area of *L. perpusilla* under duckweed weevil attack had higher than the frond area of *L. perpusilla* under control as well at 2 days after treatment (Figure 6a,b). Six days after plants were placed in the cage, the fronds of both *Lemna* species were fully expanded, and no difference was found.

## 4 DISCUSSION

In the preference and performance assays, we found that female duckweed weevils of *T. lemnae* preferred to oviposit and feed on *L. perpusilla* over *L. minor* (Figures 2a,b). The larvae on *L. perpusilla* grew larger than those on *L. minor* (Figures 2c,d). These results suggest that females of the aquatic herbivorous insect *T. lemnae* select host plants to optimize offspring performance, which supports the optimal oviposition theory; *T. lemnae* are able to choose suitable plants for their offspring (Akol et al., 2013; Lee et al., 2016; Zhang et al., 2012). Further experiments are needed to identify oviposition factors of the duckweed weevil.

Among plant defense hormones, JA plays an important role in the regulation of plant defense responses to attacks by herbivorous insects (Erb et al., 2012; Pieterse et al., 2012). Although exogenous treatment of JA increase rice resistance against rice water weevil attack (Hamm et al., 2010), it has not been tested whether endogenous JA levels of aquatic herbivore are also induced by aquatic herbivore attack. Our results show that the levels of JA was highly induced in the fronds of both *Lemna* species, *L. perpusilla* and *L. minor* by duckweed weevil, *T. lemnae* attacks (Figure 3a), which support the hypothesis that the defensive responses of free‐floating *Lemna* species to herbivorous insects is JA dependent as known in terrestrial plants. In both control and damaged *L. minor*, the levels of ABA were significantly higher than those in *L. perpusilla*. The results suggest that *L. minor* may be more sensitive to abiotic stress response than *L. perpusilla*, other than herbivore‐induced stress. It is known that ABA plays a crucial role under various environmental stress conditions including cold, drought, and salt (Erb et al., 2012). Considering both species grow primarily in summer on the water, there is a strategy to adapt to salinity conditions that may change seasonally in nature (Marcos et al., 2018). However, the levels of major flavonoids in the two *Lemna* species were not induced by duckweed weevil attack but showed significant differences in contents between two sibling *Lemna* species. Although our results suggest that *Lemna* accumulates defensive metabolites at high levels even without herbivore attack (Erhard et al., 2007), other toxic secondary metabolites may also be induced by JA when herbivores attack. To examine the defensive mechanism of two *Lemna* species in detail, we need to collect more genotypes of each species. Unique defensive mechanisms might be developed in *L. minor* and *L. perpusilla* under harsh or specific environmental conditions (e.g., interspecific competition among duckweeds) (Hart et al., 2019).

The flavonoid compositions of several duckweed species has been studied, because they have value in human health, medicine, and bioenergy (Böttner et al., 2021; Pagliuso et al., 2020; Tao et al., 2017; Wang et al., 2014). For instance, a giant duckweed, *S. polyrhiza*, contains four major flavonoids; cynaroside, orientin, apigetrin, and vitexin and the levels of those flavonoids vary in response to abiotic factors (Böttner et al., 2021). In addition, various flavanol glucoside and cycloartane glucoside have been investigated in *Landoltia punctate* (Wang et al., 2014). Another *Lemna* species, *L. gibba* has been indentified three major flavonoids, luteolin glucosides and vitexin, and this previous study showed that five genera of Lemnaceae identified different flavonoid profiling with significant contents of apigenin and luteolin derivatives (Pagliuso et al., 2020). Consistent with other duckweed studies, our results showed that the dominant flavones – isoorientin (luteolin derivative), vitexin, and isovitexin (apigenin derivatives) – are detected constitutively in *L. minor.* However, little is known about defense functions of the flavonoid compounds in duckweed species against aquatic herbivore attacks. In this study, we showed that the three major flavones protect the fronds against aquatic herbivore weevil attacks (Figure 5). In case of other macrophyte species, the submerged macrophyte *Elodea nuttalli* plant produced has been studied for functional test of some flavonoid compounds such as luteolin, apigenin, and chrysoeriol‐7‐O‐diglucuronide in response to against aquatic herbivorous Lepidoptera attacks (Erhard et al., 2007).

In this study, we show that two aquatic free‐floating *Lemna* species make different growth‐defense tradeoffs against herbivorous insect attacks. *L. perpusilla* grew faster than *L. minor* with or without herbivores (Figure 6b), while, *L. minor* accumulated more defensive substances that reduce the larval survival rates of *T. lemnae* (Figures 4 and 5). This result suggests that *L. minor* invests more energy in producing toxic metabolites rather than in promoting growth. Further studies are required to (a) show how growth‐defense tradeoffs in the two *Lemna* species in response to aquatic herbivore attack are regulated at the molecular level, (b) examine the ecological consequences of plant resource allocation, and (c) elucidate the defensive role of two *L. perpusilla*‐specific flavones: hesperetin and apigenin.

## AUTHOR CONTRIBUTIONS

**Gisuk Lee:** Conceptualization (lead); data curation (lead); formal analysis (lead); investigation (lead); methodology (lead); visualization (lead); writing – original draft (lead); writing – review and editing (equal). **Hanyoung Choi:** Data curation (supporting); formal analysis (supporting); investigation (supporting); methodology (equal). **Youngsung Joo:** Conceptualization (supporting); investigation (supporting); resources (supporting); supervision (equal); writing – review and editing (equal). **Sang‐Gyu Kim:** Funding acquisition (lead); resources (lead); supervision (lead); writing – review and editing (equal).

## CONFLICT OF INTEREST

No conflicts of interest or competing interests to report.

## Supporting information


Appendix S1

## Data Availability

The data for the study are deposited in Dryad: https://doi.org/10.5061/dryad.stqjq2c6h.
