# Supplementary material for: Flavone‐associated resistance of two Lemna species to duckweed weevil attack
Source: Ecol Evol. 2022 Nov 18;12(11):e9459. doi: 10.1002/ece3.9459 (PMC9674451; doi:10.1002/ece3.9459)
Supplement: Supplementary file 1 — Appendix S1 [file ECE3-12-e9459-s001.docx]

**Appendix**

**Supplementary Information**

**Table S1.** Compounds list used as flavonoids profiling of *Lemna* species for standards.

| **Compound name** | **Precursor ion**  **(m/z)** | **Precursor RT**  **(min)** | **Product ion**  **(m/z)** | **Polarity** | **Flavonoid type** |
| --- | --- | --- | --- | --- | --- |
|  |  |  |  |  |  |
| Catechin | 291 | 4.917 | 139, 250.2 | Positive | Flavonol |
| Epicatechin | 291 | 5.283 | 139 | Positive | Flavonol |
| Orientin | 449 | 5.814 | 299.2, 367.1 | Positive | Flavones |
| **Isoorientin** | **449** | **5.875** | **299, 329.1** | **Positive** | **Flavones** |
| **Vitexin** | **433** | **6.161** | **313, 283.05** | **Positive** | **Flavones** |
| Rutin | 611 | 6.444 | 303, 465.0 | Positive | Flavonol |
| **Isovitexin** | **433** | **6.598** | **283, 313.15** | **Positive** | **Flavones** |
| Hesperidin | 611 | 6.732 | 303, 219 | Positive | Flavanones |
| Naringin | 581 | 6.779 | 273, 339.15 | Positive | Flavanones |
| Daidzein | 255 | 8.756 | 199.1, 152.2 | Positive | Isoflavones |
| **Luteolin** | **287** | **9.426** | **153** | **Positive** | **Flavones** |
| Phloretin | 275 | 9.759 | 107, 193.05 | Positive | Chalcones |
| Narigenin | 273 | 9.879 | 153 | Positive | Flavanones |
| Genistein | 271 | 10.422 | 153, 214.95 | Positive | Isoflavones |
| **Apigenin** | **271** | **10.587** | **153, 119.15** | **Positive** | **Flavones** |
| **Hesperetin** | **303** | **10.759** | **153** | **Positive** | **Flavanones** |
| Kaempferol | 287 | 10.944 | 153 | Positive | Flavonol |
| Chalcone | 210 | 12.927 | 103.2, 131.15 | Positive | Chalcones |

Six flavonoid compounds were detected from two *Lemna* species (bold). m/z; mass to charge ration and RT; retention time.


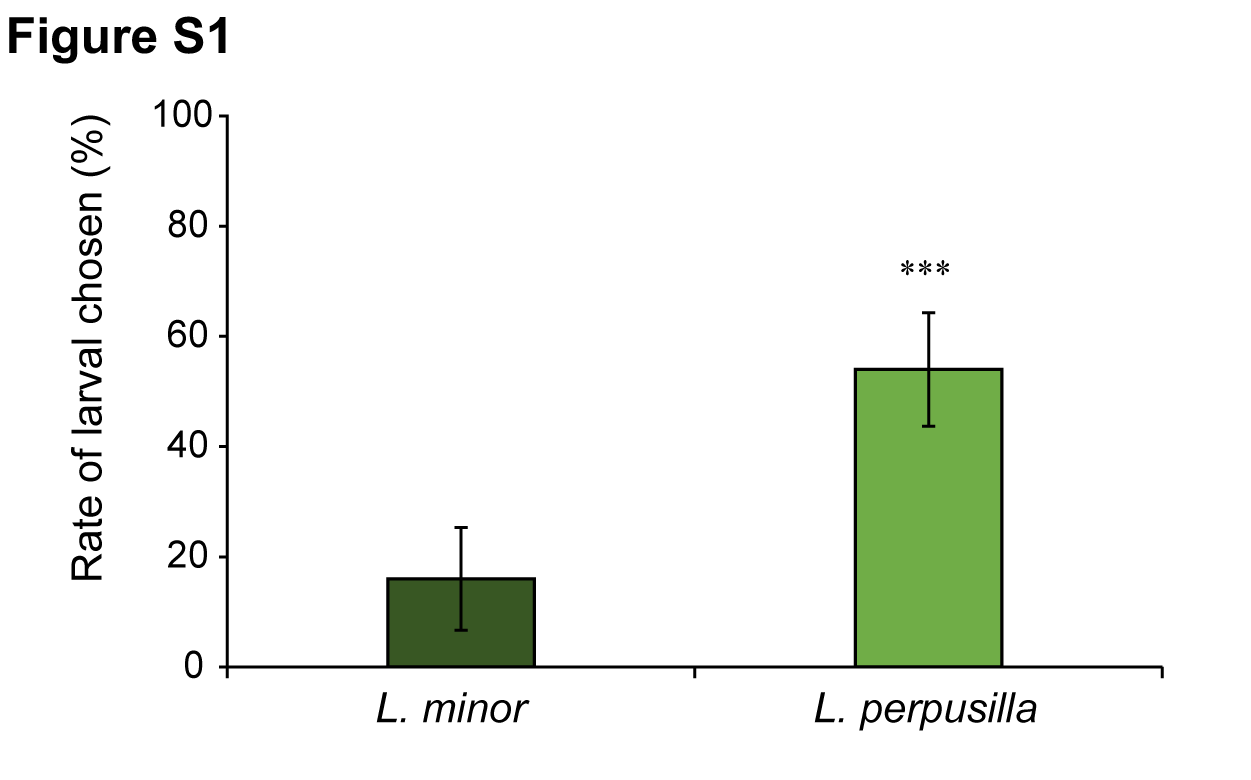


**Figure S1.** *T. lemnae* larval preference in *L. minor* versus *L. perpusilla*. Percentages (±SE) of larvae found on *L. minor* and *L. perpusilla* 1 day after release. An asterisk indicates significant differences (*G-test*, ***, *P* < 0.001, n=50).

**
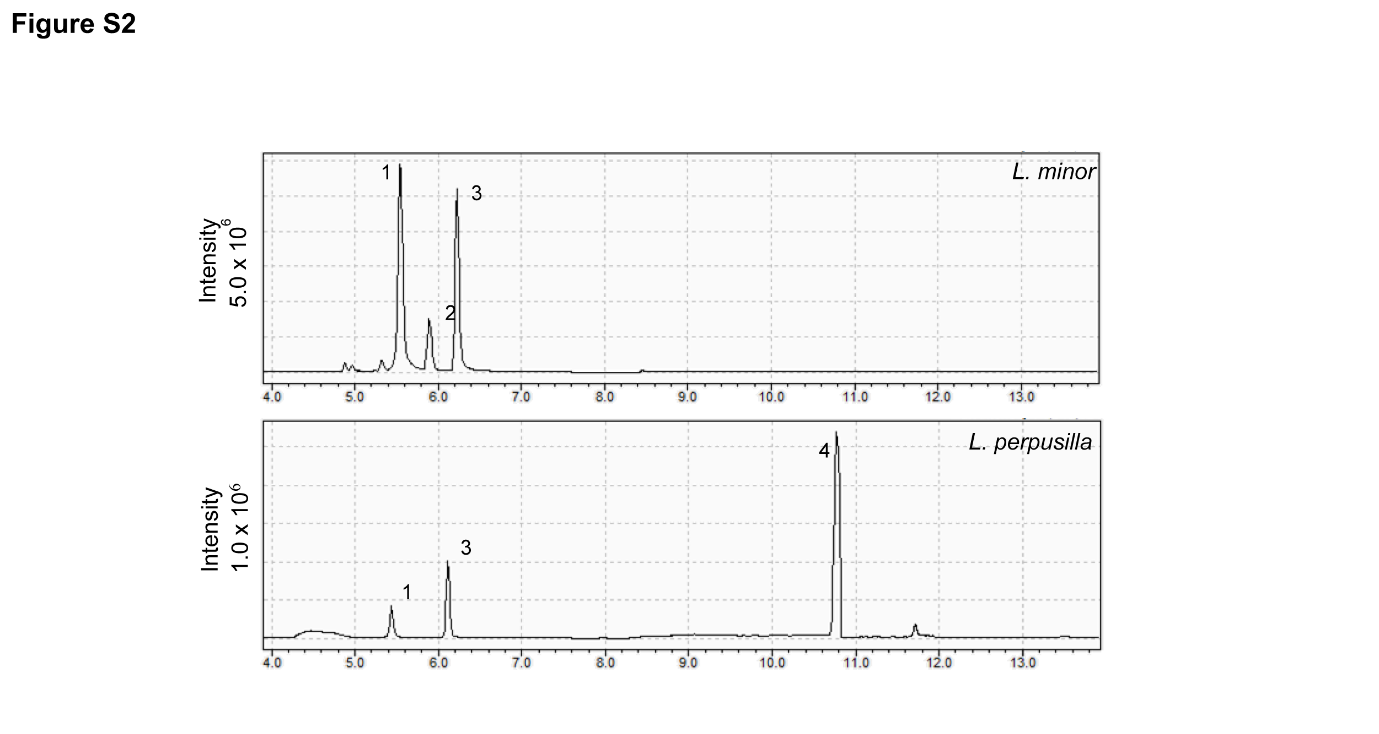
**

**Figure S2.** Chromatogram comparisons of four major flavonoid compounds (1: isovitexin, 2: vitexin, 3: isovitexin, 4: hesperetin) detected in two *Lemna* species, *L. minor* and *L. perpusilla*.

**
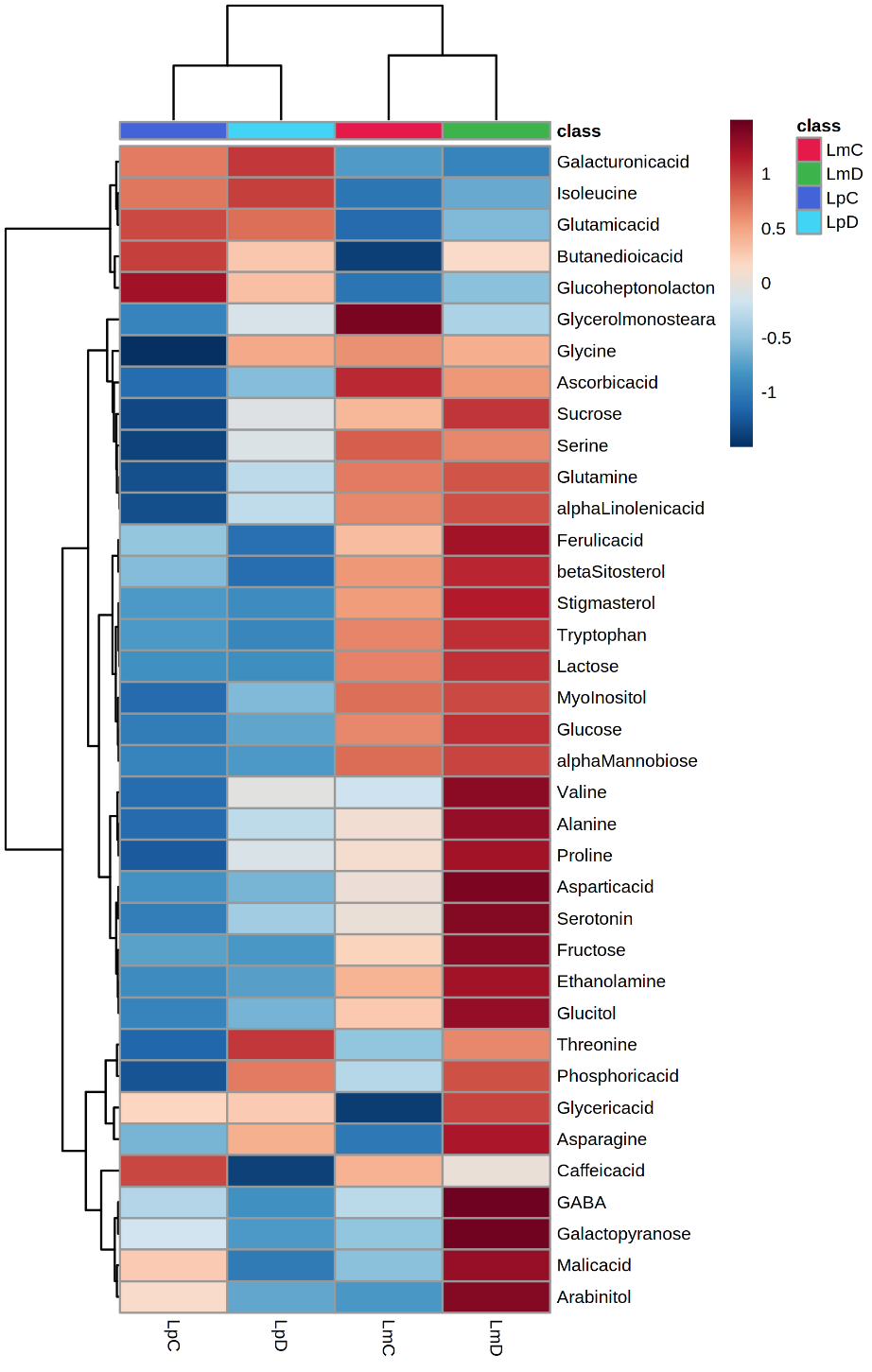
(a)**

**(b)**

**
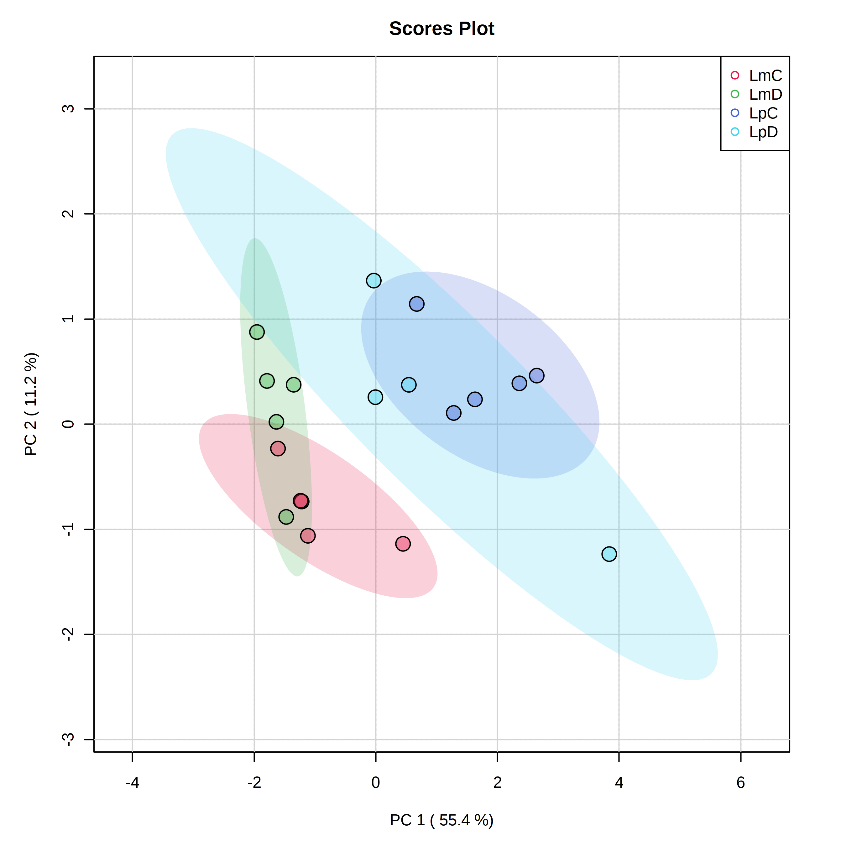
**

**Figure S3.** HeatMap using the Euclidean distance as the clustering metric of treatment relative metabolites peak intensities profiles. The heat map coloring describes the scaled metabolites peak intensities using log2foldchange (a) and principal component analysis (PCA) (b) of the primary metabolites in *L. minor* and *L. perpusilla* fronds when fronds are damaged by the duckweed weevil. LmC: *L. minor* control; LmD: *L. minor* damaged; LpC: *L. perpusilla* control; LpD: *L. perpusilla* damaged.
